# KDM3B Predicts Expression Signature, Prognosis Value, and Immune Characteristics of Cancers: A Pan‐Cancer Analysis

**DOI:** 10.1155/bmri/6916290

**Published:** 2026-04-26

**Authors:** Liping Zhu, Xiaoyan Peng, Haile Yu, Xiaolu Fan, Zhoujie Ye

**Affiliations:** ^1^ Fujian Children′s Hospital (Fujian Branch of Shanghai Children′s Medical Center), College of Clinical Medicine for Obstetrics and Gynecology and Pediatrics, Fujian Medical University, Fuzhou, Fujian, China, fjmu.edu.cn; ^2^ Medical Research Center, Fujian Maternity and Child Health Hospital, College of Clinical Medicine for Obstetrics and Gynecology and Pediatrics, Fujian Medical University, Fuzhou, Fujian, China, fjmu.edu.cn; ^3^ Fujian Key Laboratory of Women and Children′s Critical Diseases Research, Fujian Maternity and Child Health Hospital (Fujian Women and Children′s Hospital), Fuzhou, Fujian, China

**Keywords:** histone, immune checkpoint inhibitor, immune infiltration, KDM3B, pan-cancer

## Abstract

Dysregulation of histone modifications contributes to the development of cancer. The loss of methylation on histone H3 and H4 and the loss of acetylation serve as indicators of tumors. Histone demethylase KDM3B, which contains a JmjC domain, plays an important role in tumorigenesis and development by removing H3K9me1/2 methylation. It also has a significant impact on regulating different types of cancer. KDM3B′s role in prognosis and tumor formation in cancer has not been thoroughly studied. In this study, we analyzed KDM3B expression data from the TCGA database in a pan‐cancer analysis. Our results show that KDM3B is highly expressed in many cancer types and is closely associated with poor prognosis. KDM3B expression is associated with immune checkpoint activities, lymphocyte infiltration, and immune landscape changes, suggesting that KDM3B could be a target for cancer therapy.

## 1. Introduction

Epigenetics is a field concerned with gene control that does not involve changes in the underlying DNA sequence. It comprises different control techniques such as DNA methylation, histone modifications, noncoding RNAs, RNA modifications, and chromatin rearrangement [1]. Especially, histone modifications such as methylation and demethylation mediated by histone methyltransferases (HMTs) and histone demethylases (HDMs) are important components of this epigenetic process. These alterations act cooperatively to regulate DNA repair, gene transcription, cell cycling, and cellular stress responses, and occur in many biological contexts such as growth, development, and aging [2–4]. Histone methylation: Histone methylation is a type of posttranslational histone modification that refers to the addition of a methyl group to arginine (R) and lysine (K) residues present in the N‐terminal tails of histone H3 and histone H4. It aids in recruiting regulatory proteins that function to either activate or repress gene expression [5]. For example, methylation of lysine residues on histone H3 at H3K4 or H3K36 corresponds to active transcription; on the other hand, methylation of H3K9 and H3K27 is associated with gene expression repression [6–8]. Therefore, explaining how different kinds of histone modification recognition occur will help us better understand why epigenetically regulated regions activate certain genes.

KDM3B, a specific H3K9me1/me2 demethylase in the JMJD family that possesses the JmjC domain, can remove either mono‐ or dimethyl groups from H3K9 [9]. Initially recognized as 5qNCA and mapped to chromosome 5q31, which is also a frequent deletion site in myelodysplastic syndromes (MDS), KDM3B contains a zinc finger domain, a JmjC domain, and a nuclear localization domain [10, 11]. The JmjC domain, which is characteristic of chromatin remodeling proteins, confers HDM activity [12]. The expression of KDM3B across many different types of human tissues, such as the heart, skeletal muscle, kidney, placenta, liver, and both CD34^+^ lymphocytes as well as multiple AML cell lines, highlights the fact that it plays an important role in biological development [10]. In the past, some studies have shown that KDM3B plays many biological roles, such as the development of certain body parts, angiogenesis, hematopoiesis, regulation of gene expression during DNA replication, maintenance of chromosome number after DNA replication is completed, cell differentiation, spermatogenesis, and maintenance of estrogen hormone balance [9, 13–17].

KDM3B displays a conditional twofold function during tumorigenesis. KDM3B works like a brake on tumors in some contexts: It can slow down myeloid leukemia cells, colorectal cancer cells, breast cancer cells, and prostate cancer cells [9, 18, 19]. On the other hand, it has also been seen as an oncogenic player in the case of ALL (acute lymphoblastic leukemia), since KDM3B′s actions are linked with the chromatin accessibility of regions around the gene where LMO2 is located [9]. KDM3B thus becomes an interesting therapeutic target when investigating cancer treatment. Due to its altered expression across different cancer systems and because it may have both tumor‐suppressive and tumor‐promoting activities, it represents a potential drug target. Henceforth, we performed a pan‐cancer study to conduct a comprehensive investigation into alterations in the level of KDM3B expression across many different forms of cancer and to examine whether there is a relationship between KDM3B expression levels and survival time, as well as to determine whether it can serve as an indicator predicting the outcome of immunotherapy treatment.

This study uses transcriptomic information from both The Cancer Genome Atlas (TCGA) pan‐cancer dataset and the GTEx dataset to assess how KDM3B expression varies among different cancers, to evaluate its prognostic value, and to determine whether it is associated with treatment response. Univariate Cox proportional hazards regression models (PHRMs) and Kaplan–Meier survival analyses (KM SAs) were performed to assess the prognostic value of KDM3B in multiple cancer types. In addition, gene set enrichment analysis (GSEA) was conducted to identify tumor‐associated signatures related to KDM3B expression. Several quantitative methods were used to examine immune cell infiltration levels and the relationships between these levels and KDM3B expression across various cancer types. Additionally, tumor mutational burden (TMB) and microsatellite instability (MSI) were evaluated to better define the potential of KDM3B to predict responses to immunotherapy.

## 2. Methods and Materials

### 2.1. Data Source and Processing

This study utilizes the pan‐cancer cohort from TCGA database. Tumor tissue transcriptome data were sourced from the TCGA pan‐cancer cohort, whereas transcriptome data for normal human tissues were obtained from the Genotype–Tissue Expression (GTEx) project [20]. The expression profiles were converted to transcripts per kilobase million (TPM) format. We used log2(TPM + 1)‐formatted data for further analysis to avoid the situation where the TPM value is 0. Gene Expression Profiling Interactive Analysis 2 (GEPIA2) (http://gepia2.cancer-pku.cn/#dataset) was used to analyze the expression differences of KDM3B across 34 different tumor types and the corresponding normal tissues [21, 22].

### 2.2. Genomic Alteration Analysis of KDM3B in Human Cancers

For genomic alteration analysis, cBioPortal (http://www.cbioportal.org/) provided access to a wide array of data, including DNA copy number, mRNA and miRNA expression, nonsynonymous mutations, protein levels, phosphoprotein levels (RPPA), clinical data, and DNA methylation data [23]. This study used cBioPortal to examine the genomic alterations (mutations, structural variations, amplifications, deep deletions, and genetic alterations) and the frequency of multiple alterations of KDM3B across various cancers, which were visualized through graphical representations.

### 2.3. KDM3B Subcellular Localization and Interacting Protein Network

The Human Protein Atlas (HPA) (https://www.proteinatlas.org/) provided detailed insights into protein expression differences in normal and tumor tissues [24]. We observed the subcellular localization of KDM3B in A‐431 (human epidermoid cancer cell line) and U251 (human astroglial cell line) using immunofluorescence staining from the HPA database. Additionally, the STRING database (https://string-db.org/) was utilized for visualizing protein–protein interactions (PPIs) and for information on protein families, pathways, and subcellular localizations. We downloaded the PPI data of KDM3B from STRING, analyzed its potential interacting protein network, and presented the findings pictorially.

### 2.4. Prognostic Analysis

The prognostic significance of KDM3B for each type of malignant neoplasm was assessed using the Kaplan–Meier method and univariate Cox regression analysis [25]. The continuous expression data of KDM3B were used in univariate Cox regression analyses, whereas the bivariate expression levels of KDM3B were employed to perform Kaplan–Meier curve analyses, with the cutoff point selected using the “surv_cutpoint” function from the “survminer” package in R. Subsequently, the log‐rank *p* values and hazard ratios (HR) with 95% confidence intervals (CI) were calculated. The results were consolidated and presented in the form of a heatmap.

### 2.5. Clinical Staging and Gene Expression Analysis of Cancer

A standardized pan‐cancer dataset was downloaded from the UCSC database [26], and the expression differences of KDM3B in different cancers at various clinical stages, including tumor (T), node (N), and metastasis (M) stages, were analyzed using R software (Version 3.6.4). The differences between two groups were evaluated using unpaired Student′s *t*‐tests, whereas analysis of variance (ANOVA) was employed for multigroup sample comparisons.

### 2.6. GSEA

Based on differentially expressed genes (DEGs) between high‐ and low‐expression groups of KDM3B, pan‐cancer GSEA analysis was performed in 33 cancer types using R software′s ClusterProfiler 4.0 package. The pathways for enrichment analysis were derived from the Hallmark gene set, which encompasses 50 well‐defined biological states and processes (available at https://www.gsea-msigdb.org/gsea).

### 2.7. Immune Cell Infiltration Analysis of KDM3B

TIMER, a comprehensive resource for systematic analysis of immune infiltrates across diverse cancer types, was utilized. This webserver estimates the abundances of immune infiltrates through multiple deconvolution methods, enabling dynamic, high‐quality figure generation for exploring tumor immunological, clinical, and genomic features [27, 28]. KDM3B‐related immune cell infiltration correlations were obtained from the TCGA pan‐cancer project in the TIMER2.0 database (http://timer.cistrome.org/). We downloaded the data, which allows identification of KDM3B associations with immune cell infiltrates linked to TCGA pan‐cancer project data. The data included CD8^+^ T cells, CD4^+^ T cells, B cells, Treg cells, myeloid dendritic cells, and macrophages, which were visualized in a heatmap using the R package ggplot2.

### 2.8. Analysis of KDM3B Immune Regulatory Gene

The TCGA, TARGET, and GTEx databases (PANCAN), with the GTEx dataset in PANCAN (*N* = 19,131; *G* = 60,499), were used for extraction of KDM3B gene expression and 150 markers for five pathways, including chemokines (41), receptors (18), major histocompatibility complex (MHC) (21), immunoinhibitors (24), and immunostimulators (46). Data were then filtered to include only primary solid tumors and bone marrow– and peripheral blood‐derived cancers; normal samples were excluded. Log2 transformation was applied to each expression value as log2(*x* + 0.001), and Pearson correlation analysis was performed between KDM3B and immune pathway marker genes.

### 2.9. Culture and Construction of Cell Lines

Renal cancer cell line Caki‐1 and human colon cancer cell line HCT‐116 were both obtained from iCell Bioscience Inc., Shanghai, China. All cell lines were authenticated by STR profiling and confirmed to be free of mycoplasma contamination. Caki‐1 and HCT‐116 cells were cultured in McCoy′s 5A medium supplemented with 10% fetal bovine serum (Gibco, #6123139) in a humidified incubator at 37°C with 5% CO_2_. Lentiviruses expressing shKDM3B were synthesized and produced by Gemma Pharmaceutical Technology Co., Ltd., Shanghai. Cells were seeded in six‐well plates. When the cell density reached 50%, shKDM3B lentivirus was added and incubated for 8 h, after which the supernatant was removed and replaced with fresh medium. At 48 h postlentiviral infection, puromycin was added for 1 week of selection to establish Caki‐1*^shKDM3B^* and HCT‐116*^shKDM3B^* stable cell lines.

### 2.10. Cell Proliferation and Colony Formation Experiments

Cell proliferation was detected using the CCK‐8 assay (Dojindo, #HY‐K0301). Briefly, cells in the logarithmic growth phase (5 × 10^3^) were seeded into 96‐well plates with three replicate wells per group. CCK‐8 working solution was added at the same time each day and incubated for 2 h. The optical density (OD) at 450 nm was measured using a microplate reader (BioTek Cytation 1, United States), and the proliferation curve was plotted. For the colony formation assay, cells (1 × 10^3^) were seeded into 12‐well plates, and the experiment was repeated three times. After 2 weeks of culture, cells were fixed with tissue fixative for 20 min and stained with 0.1% crystal violet (Solarbio, #C8470) for 10 min. Excess staining solution was removed, and photographed for documentation.

### 2.11. Transwell Migration and Invasion Assays

Cells in the logarithmic growth phase were collected, and the cell suspension was adjusted to a density of 1 × 10^6^ cells/mL using medium containing 5% serum. Two hundred microliter of the cell suspension was added into migration chambers (Corning, #08722022) or Matrigel‐precoated invasion chambers. Six hundred microliter of medium containing 20% serum was added to the lower chambers. After 24 h, nonmigrated cells were removed, and cells on the membranes were fixed with methanol and stained with crystal violet. Positive cells in five independent fields were imaged under a microscope and quantitatively analyzed using ImageJ software. The experiment was repeated three times in total.

### 2.12. Cell Cycle Assay

Cells from each group (1 × 10^6^) were harvested and fixed overnight in 80% ethanol, followed by centrifugation at 2000 rpm for 5 min. The cells were washed with precooled PBS to remove the fixative. Subsequently, the cells were incubated with RNase A at 37°C for 30 min, stained with 200 *μ*L of 50‐*μ*g/mL propidium iodide (PI) (Elabscience, China, #E‐CK‐A351) for 30 min, and analyzed by flow cytometry (BD LSRFortessa, United States) within 1 h. This experiment was repeated three times in total.

### 2.13. Wound Healing Assay

Four parallel lines were drawn at equal distances on the bottom of 12‐well plates in advance. Cells in the logarithmic growth phase (4 × 10^5^) were seeded, with three replicate wells per group. When cells adhered to the wall and reached 80% confluence, a horizontal scratch was created in the cell monolayer using a pipette tip. The scratched area was washed with PBS, and medium containing 5% serum was added. Wound healing progression was recorded at 0, 24, and 72 h.

### 2.14. Statistical Analyses

Correlations between variables were analyzed using Pearson or Spearman correlation coefficients. Comparisons between groups of normally distributed continuous variables were performed using the *t*‐test, whereas nonnormally distributed variables were analyzed using the Mann–Whitney *U* test. Prognostic survival curves for categorical variables were plotted using the Kaplan–Meier method, and comparisons between groups were performed using the log‐rank test. All statistical tests were two‐sided, and *p* < 0.05 was considered statistically significant. Bioinformatics and clinical data statistical analyses were performed using R software (Version 3.6.3). Molecular and cellular experimental data were analyzed using GraphPad Prism software. Comparisons between two groups of normally distributed data were performed using the two‐tailed unpaired Student′s *t*‐test, and comparisons among multiple groups were performed using one‐way or two‐way ANOVA. Data are presented as the mean ± standard deviation (mean ± SD).

## 3. Results

### 3.1. Expression, Genetic Alteration, and Protein Characteristics of KDM3B in Human Tumors

**Figure 1:**
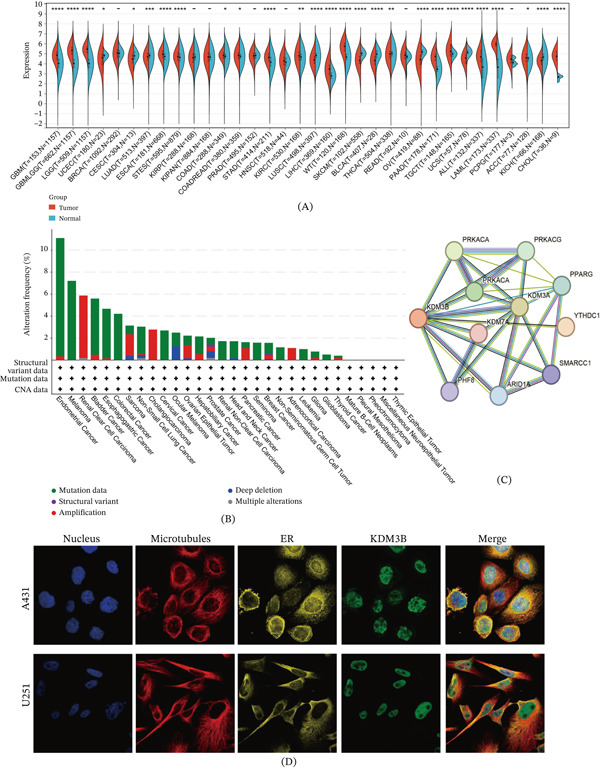
Basic information of KDM3B. (A) Based on the TCGA and GTEx datasets, the expression levels of KDM3B in different tumor types and normal tissues were investigated. (B) Analysis of the alteration frequency of KDM3B in pan‐cancer research according to the cBioPortal database. (C) The protein–protein interaction (PPI) network presents proteins interacting with KDM3B. (D) Immunofluorescence images of KDM3B protein, nucleus, endoplasmic reticulum (ER), microtubules, and the merged images in A431 and U251 cell lines.

We integrated data from the TCGA and GTEx databases to scrutinize the transcriptional expression levels of KDM3B across different cancer types. The results showed that KDM3B expression was significantly upregulated in 17 of 34 common cancers compared with normal tissue: GBM, GBMLGG, LGG, ESCA, STES, COAD, COADREAD, STAD, KIRC, LIHC, WT, PAAD, TGCT, ALL, LAML, ACC, and CHOL. In comparison, low KDM3B expression was observed in 10 tumors: UCEC, CESC, LUAD, LUSC, SKCM, BLCA, THCA, OV, UCS, and KICH (Figure 1A). Utilizing the cBioPortal tool, our analysis uncovered significant diversity and frequency of genetic variations in the KDM3B gene. We evaluated the transcriptional expression of KDM3B in the TCGA cohort with varying copy numbers, identifying 320 variants of uncertain significance (VUS) across multiple tumors. The highest mutation frequency was noted in endometrial cancer (11.91% of 529 cases), comprising 11.72% mutations and 0.19% amplifications. Other cancers with notable frequencies included melanoma (7.21%), colorectal cancer (4.21%), cervical cancer (2.69%), and renal nonclear cell carcinoma (1.72%), along with others listed in Figure 1B. To gain deeper insights into the KDM3B protein, we examined immunofluorescence images from the HPA. From these images, the localization of KDM3B was observed mainly in the nucleus, with most of the signal concentrated in the nuclei of A431 and U251 cells, as seen in Figure 1D. Minimal cytosolic expression aligns with KDM3B′s role in nuclear HDM. The PPIs retrieved from the STRING database primarily associate KDM3B with KDM3A, YTHDC1, PRKACB, PRKACG, and PPARG. These proteins are also involved in modifying histones, reducing methylation, and altering chromatin structure (Figure 1C). Multidatabase analyses comprehensively described KDM3B′s expression map, genetics, and molecular interactions in various cancers. The data show that KDM3B transcription levels and genomic alterations vary significantly depending on the cancer type.

### 3.2. Clinical Prognostic Significance of KDM3B Across Pan‐Cancer

**Figure 2:**
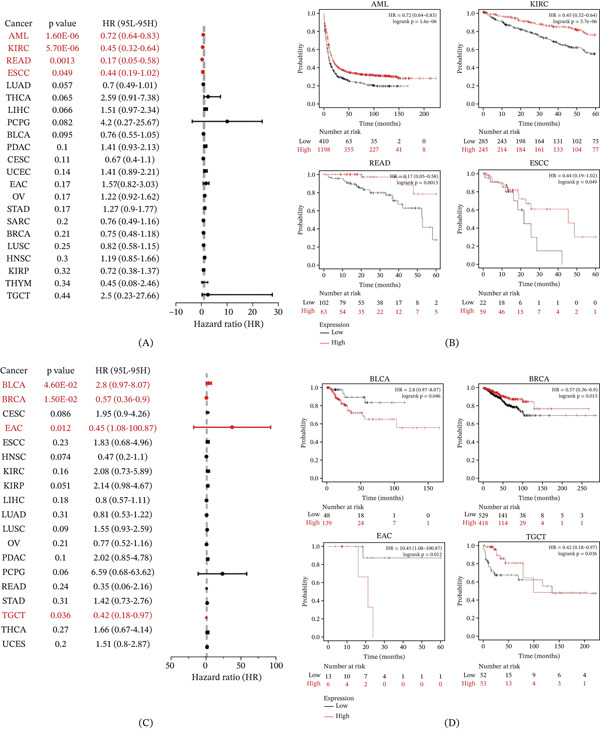
Relationship between KDM3B and prognosis in pan‐cancer. (A) The forest plot shows the role of KDM3B in OS in cancer based on univariate Cox regression analysis. (B) Kaplan–Meier overall survival curves of KDM3B in AML, KIRC, READ, and ESCC. (C) The forest plot shows the role of KDM3B in RFS in cancer based on univariate Cox regression analysis. (D) Kaplan–Meier recurrence‐free survival curves of KDM3B in BLCA, BRCA, EAC, and TGCT.

Overall survival (OS) is a key efficacy endpoint in oncology clinical trials and is the preferred measure for assessing patient survival. Using TCGA data, we categorized KDM3B expression into high‐ and low‐expression groups and examined its prognostic relevance across different cancers. We found that KDM3B expression was a statistically significant protective factor in AML, KIRC, READ, and ESCC, with higher expression levels associated with longer OS (Figure 2A). Specifically, upregulation of KDM3B was associated with extended OS in AML (HR = 0.72, [95% CI, 0.64–0.83], *p* < 0.001), KIRC (HR = 0.45, [95% CI, 0.32–0.64], *p* < 0.001), READ (HR = 0.17, [95% CI, 0.05–0.58], *p* = 0.0013), and ESCC (HR = 0.44, [95% CI, 0.19–1.02], *p* = 0.049) (Figure 2B). Similar to the Kaplan–Meier study, KDM3B knockdown accelerates the cell cycle, prevents proper granulocyte differentiation, and promotes the growth of APL NB4 cells [29]. Recurrence‐free survival (RFS), a critical endpoint for tumor clinical studies, refers to the time from complete response posttreatment to recurrence or follow‐up termination. KDM3B expression exerted differential effects on RFS across tumors (Figure 2C,D): High KDM3B expression was a risk factor associated with shorter RFS in BLCA and EAC, whereas it functioned as a protective factor linked to longer RFS in BRCA and TGCT.

**Figure 3:**
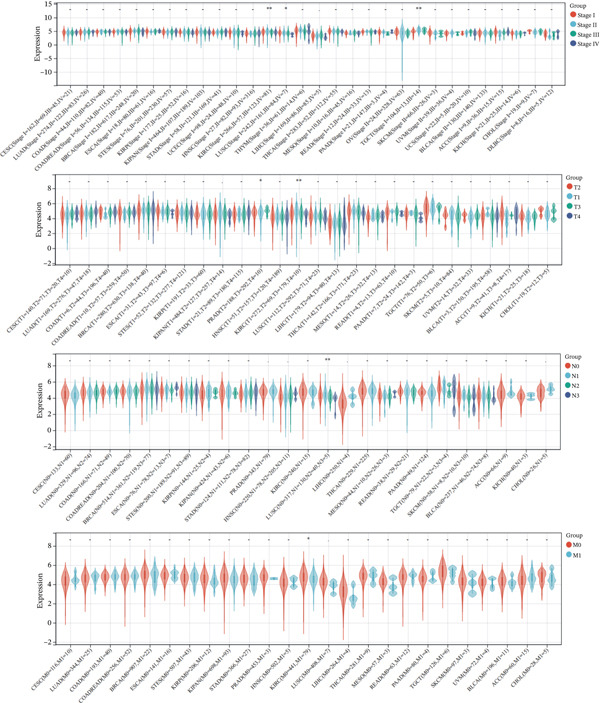
The difference of KDM3B in different pathological stages of tumor, including stage. tumor (T), node (N), metastasis (M).

We extracted the expression data of KDM3B at different tumor stages from the UCSC database. Figure 3 illustrates the variations in KDM3B expression across various stages of in situ cancer in KIRC, TGCT, and LUSC. It is important to note that KDM3B expression in the tumor (T) staging system varied in cases of KIRC and PRAD. Furthermore, disparities were observed in the lymph node (N) staging in LUSC and in the metastasis (M) staging in KIRC. KDM3B plays a prognostic role in predicting cancer outcomes, although its functions appear to be complex and vary depending on the type of cancer. Future research should focus on elucidating the role of the KDM3B protein in cancer cells.

### 3.3. Gene Enrichment Analysis of KDM3B

**Figure 4:**
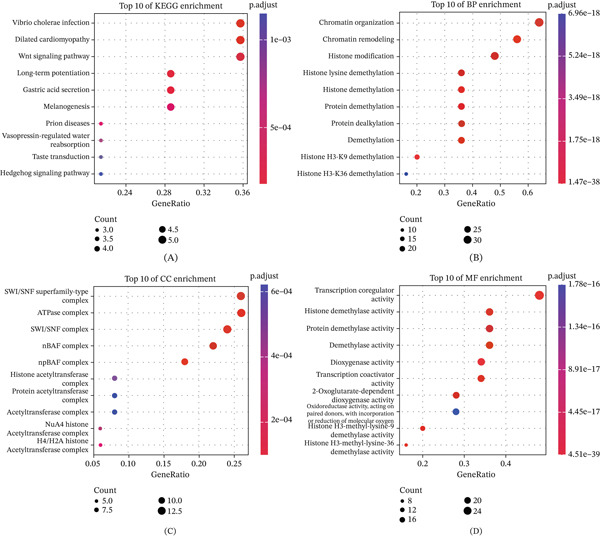
KDM3B‐related gene enrichment analysis. (A–D) Enrichment analysis of KDM3B and its most frequent neighboring genes. KDM3B‐binding and interacting genes were used to perform KEGG pathway and Gene Ontology (GO) enrichment analyses.

Using STRING database data, we identified the Top 10 proteins interacting with KDM3B and constructed a PPI network. To elucidate the functional implications of KDM3B interactions, we performed KEGG pathway and Gene Ontology (GO) enrichment analyses. KEGG pathway analysis indicated that KDM3B may be involved in the Wnt signaling pathway, which is essential for cell growth (Figure 4A). GO enrichment analysis revealed that genes interacting with KDM3B are primarily engaged in biological processes (BP), including chromatin organization, chromatin remodeling, histone modification, histone lysine demethylation, and specifically histone H3‐K9 demethylation (Figure 4B,C). These pathways are predominantly associated with histone epigenetic modification. Further analysis uncovered a tight association between KDM3B and chromatin remodeling activities, whereas molecular function (MF) category analysis demonstrated the relationship between KDM3B and multiple molecular binding capacities (Figure 4D).

**Figure 5:**
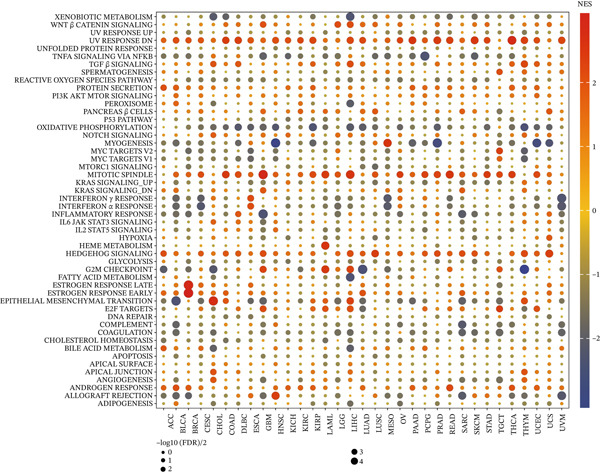
Hallmark gene set enrichment analysis (GSEA) of KDM3B in pan‐cancer. The size of the circle represents the false discovery rate (FDR) value of each enriched item, and the color represents the normalized enrichment score (NES) of each enrichment item.

To investigate the BP influenced by KDM3B expression in cancer, we performed DEG analysis by comparing high‐ and low‐KDM3B expression groups across various cancer types. It should be noted that immune pathways, particularly IFN‐*α* and IFN‐*γ*, were prominently represented in the tumors of BLCA, CESC, ESCA, MESO, LGG, READ, and UVM. This suggests that changes in KDM3B expression significantly impact immune pathways in these tumors, thereby opening new opportunities for therapies based on immunotherapeutic approaches (Figure 5). Furthermore, the role of KDM3B appears to be crucial in remodeling chromatin within the tumor immune microenvironment and in modulating ligand–receptor interactions between cancer cells and immune cells. Interestingly, a negative correlation has been observed between KDM3B expression and oxidative phosphorylation pathways in various cancers, highlighting its significant role in the metabolic energy processes of tumors. The integrative analysis of KDM3B‐interacting proteins, functional enrichment, and differential gene expression across various cancers reveals that KDM3B plays a significant role in the regulation of epigenetic mechanisms, Wnt signaling, and immune‐related pathways in cancer. Its expression is linked to chromatin organization within the immune microenvironment and oxidative phosphorylation, highlighting its potential role in cancer immunotherapy and metabolic processes.

### 3.4. Immune Cell Infiltration Analyses of KDM3B Across Cancers

**Figure 6:**
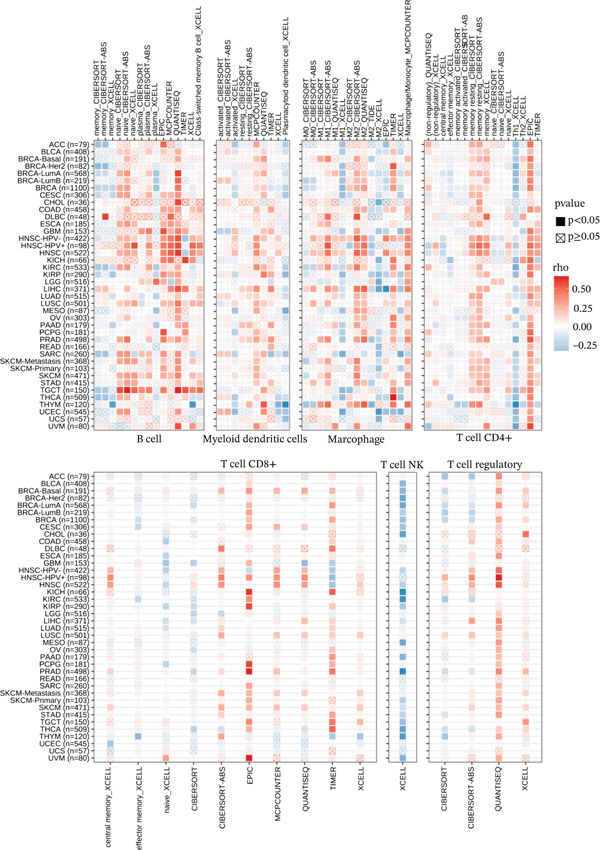
Correlation between KDM3B expression and lymphocyte infiltration. The infiltrated lymphocyte analysis included CD8^+^ T cells, CD4^+^ T cells, B cells, regulatory T cells (Tregs), myeloid dendritic cells, and macrophages.

From the results of GSEA data analysis, we investigated whether there is a relationship between KDM3B expression levels and the level of infiltrating immune cells within tumors. Using the TIMER2.0 database, we analyzed whether KDM3B was linked to different kinds of immune cells involved in tumor immune infiltration. We found that B cells, myeloid dendritic cells, macrophages, CD4^+^ T cells, CD8^+^ T cells, NKT cells, and total T cells were related to lymphocyte infiltration and immune therapy responses in many cancers (Figure 6). In general, KDM3B exhibits a positive correlation with the infiltration levels of B cells, myeloid dendritic cells, macrophages, CD4^+^ T cells, and other cell types across various tumor types, including head and neck squamous cell carcinoma (HNSC), both HPV‐negative (HNSC‐HPV−) and HPV‐positive (HNSC‐HPV+). Additionally, in testicular germ cell tumors (TGCT), KDM3B demonstrates a significant association with B cell infiltration. Generally, KDM3B exhibits a positive correlation with the infiltration levels of B cells, myeloid dendritic cells, macrophages, CD4^+^ T cells, and other cell types across various cancer types, including HNSC both with and without HPV infection (HNSC‐HPV− and HNSC‐HPV+). In addition, TGCT, as an example of TGCT, also shows a strong correlation between KDM3B expression and B cell infiltration. However, the correlation trends for other cell types were not markedly different, which may be due to differences in the extent of immune cell infiltration among different tumors. Overall, the analysis indicated that there was a relationship between KDM3B expression levels and immune cell infiltration within tumor sites.

### 3.5. KDM3B and Immune‐Related Regulatory Genes in Pan‐Cancer

**Figure 7:**
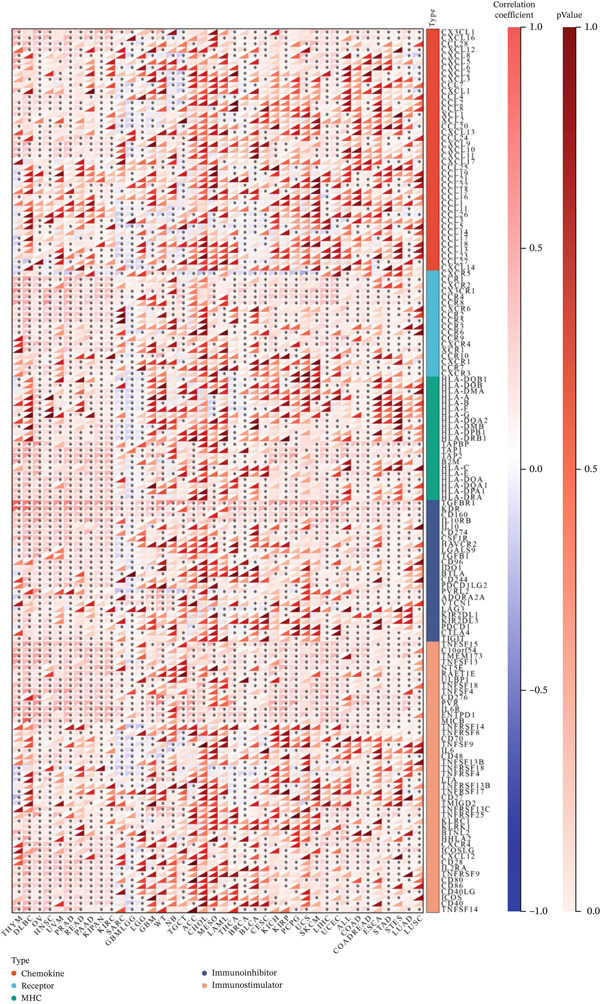
Analysis of KDM3B and immunomodulatory genes in pan‐cancer. The analysis mainly included chemokines, chemokine receptors, major histocompatibility complex (MHC), immunoinhibitors, and immunostimulators.

We conducted an analysis of the differential expression of genes associated with immune modulation linked to KDM3B. These genes are categorized into five components: chemokines, chemokine receptors, the MHC, immune inhibitors, and immune stimulators. As shown in Figure 7, KDM3B expression in THYM, DLBC, OV, HNSC, KIPAN, and KIRC is positively correlated with immune‐related genes across various cancer types. In sarcoma (SARC) and lower‐grade glioma (GBMLGG), KDM3B exhibits a negative correlation with most genes associated with immune regulation. The key protein KDM3B is significantly associated with important markers involved in regulating immune responses, such as programmed cell death protein 1 (PDCD1) (also known as PD‐1), programmed cell death ligand 1 (PD‐L1), and cytotoxic T lymphocyte‐associated protein 4 (CTLA4), across various cancer types. KDM3B expression is correlated with PD‐L1 expression in 29 types of tumors, including THYM, DLBC, OV, and HNSC; with PD‐1 expression in 20 types of tumors, such as LUSC, STAD, and COAD; and with CTLA‐4 expression in 19 types of tumors, including OV, HNSC, and LGG. To date, no direct effects of KDM3B on immune checkpoint therapy have been reported in the existing literature.

### 3.6. Correlation of KDM3B with TMB and MSI

We have examined the impact of TMB on PD‐1 and PD‐L1 inhibitor efficacy and found that it is a reliable predictor of immunotherapy outcomes in numerous clinical studies. TMB serves as a biomarker to predict the effectiveness of immunotherapy in certain cancers [30, 31]. A characteristic feature of tumors, compared with normal tissue, is the occurrence of new microsatellite alleles, resulting from the insertion or deletion of repeat units at microsatellite sites. This phenomenon, known as MSI, arises due to functional defects in DNA mismatch repair within tumor tissues. MSI, associated with these DNA mismatch repair deficiencies, serves as a crucial clinical tumor marker [32, 33].

**Figure 8:**
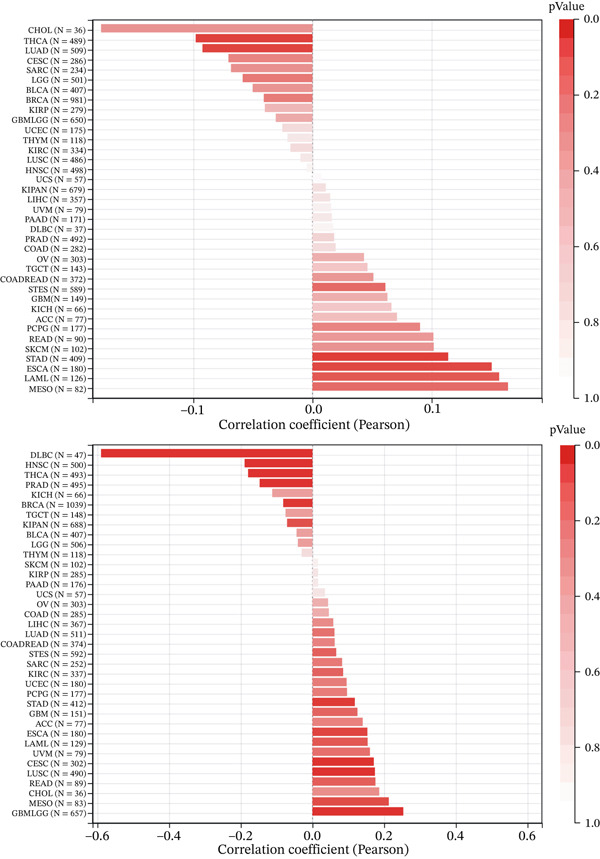
Analysis of KDM3B with tumor mutational burden and microsatellite instability in pan‐cancer.

To investigate the role of KDM3B in predicting outcomes of immunotherapy, we analyzed its association with two well‐known indicators of immune therapy efficacy: TMB and MSI. Our findings suggest that KDM3B expression in STAD and ESCA is positively correlated with TMB, whereas a negative correlation with TMB was observed in THCA and LUAD. Furthermore, KDM3B expression in GBMLGG, LUSC, CESC, and STAD demonstrates a positive correlation with MSI, whereas it exhibits a negative correlation with MSI in DLBC, HNSC, THCA, PRAD, and BRCA (Figure 8). In summary, the analysis of the relationship between KDM3B expression and these two key biomarkers for predicting immunotherapy efficacy (TMB and MSI) across multiple cancer types revealed distinct correlation patterns for each cancer type. These findings suggest a potential role for KDM3B in predicting the effectiveness of immune checkpoint inhibitor (ICI) therapies.

### 3.7. Deletion of KDM3B Inhibits KIRC and COAD Proliferation and Metastasis

**Figure 9:**
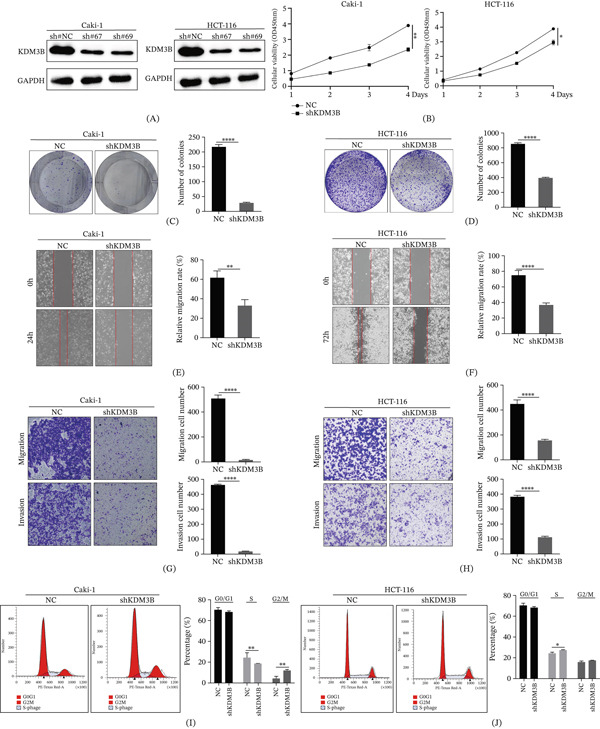
Deletion of KDM3B inhibits proliferation and metastasis of KIRC and COAD cells. (A) The expression level of KDM3B was knocked down in Caki‐1 and HCT‐116 cells. (B) CCK‐8 assays were performed to determine the proliferation of Caki‐1 and HCT‐116 cells. (C, D) Colony formation assays of KDM3B‐knockdown Caki‐1 and HCT‐116 cells. (E, F) Cell migration ability was reduced after KDM3B knockdown. (G, H) The abilities of invasion and migration were weakened after KDM3B knockdown in Caki‐1 or HCT‐116 cells. (I, J) KDM3B knockdown in Caki‐1 or HCT‐116 cells resulted in cell cycle arrest.

To further elucidate the precise biological function of KDM3B, we investigated its impact on cell growth in the clear cell renal carcinoma Caki‐1 and colorectal cancer HCT‐116 cell lines. Analysis of TCGA databases revealed that KDM3B expression is upregulated in tumor tissues of KIRC and COAD. We used lentivirus to reduce KDM3B expression in Caki‐1 and HCT‐116 cells (Figure 9A). Cell proliferation and colony formation assays demonstrated that when KDM3B expression was downregulated in Caki‐1 or HCT‐116 cells, CCK‐8 assays revealed a reduction in cell proliferation (Figure 9B), and the colony formation capacity was also inhibited compared with the NC group (Figure 9C,D). The results of the cell scratch assay demonstrated a significant reduction in the migration rate of Caki‐1 or HCT‐116 cells when KDM3B expression was downregulated (Figure 9E,F). Furthermore, after KDM3B downregulation, the invasive and migratory capabilities of Caki‐1 or HCT‐116 cells were significantly reduced (Figure 9G,H). Compared with the NC, KDM3B downregulation prolonged the cell cycle duration in Caki‐1 or HCT‐116 cells (Figure 9I,J). At the cell line level in vitro, the absence of KDM3B inhibits proliferation and migration of Caki‐1 and HCT‐116 cells, reduces cancer cell aggressiveness, and suppresses their biological functions.

The findings of this study support the potential of KDM3B as a biomarker for immune checkpoint therapy response in cancer. However, to confirm its role as a biomarker in cancer treatment and its impact on specific biological functions, further in‐depth studies are required in both basic research and clinical contexts.

## 4. Conclusion

Histone proteins are essential for maintaining DNA structure, protecting genetic information, and regulating gene expression [34, 35]. The amino‐terminal domain of histones extends outside the nucleosome, allowing interactions with regulatory proteins and DNA. Key histone modifications, such as methylation, phosphorylation, acetylation, crotonylation, ubiquitination, glycosylation, and ADP ribosylation, regulate gene expression [36]. Imbalances in these modifications, especially the loss of methylation and acetylation on histones H3 and H4, are associated with tumorigenesis [37, 38]. HDM KDM3B is involved in the regulation of histone lysine methylation and also influences the formation of gene promoter complexes. This regulation affects cell cycle progression as well as proliferation and apoptosis, which can either promote or inhibit tumor development [39, 40]. KDM3B is particularly important for the tumorigenicity and survival of human colorectal cancer stem cells, mainly by activating genes related to the Wnt signaling pathway [41].

Although KDM3B is known to play a significant role in cancer, its specific function in different human tumors requires further investigation [42–44]. In this study, we analyzed KDM3B gene alterations, along with mRNA and protein expression levels, using data from the TCGA and GTEx cohorts. We also conducted enrichment analysis on genes that interact with or are related to KDM3B to gain a deeper understanding of how KDM3B expression is linked to cancer progression.

From our analysis, we concluded that in four cancer types, there is abnormal overexpression of KDM3B, and most of these cancers had genomic changes of approximately 5% or less. The exceptions were endometrial cancer and melanoma, both of which exhibited a higher frequency of these genetic alterations. Regarding KDM3B‐differential genes, KEGG and GO enrichment analyses indicated regulation of cell development via the Wnt signaling pathway. This supports previous findings that the KDM3 family influences transcription of genes in the Wnt/*β*‐catenin pathway, which are vital for the tumorigenicity and survival of colorectal cancer stem cells [45]. Additionally, our GO enrichment results highlighted histone methylation and chromatin remodeling, suggesting potential areas for further investigation into the epigenetic regulation of the Wnt pathway in cancer development.

We also investigated the relationship between KDM3B expression and patient outcomes. We found that KDM3B acted as a protective factor for survival, although its expression varied across different tumors during tumor progression. Interestingly, KDM3B was a protective factor for OS in four tumor types, whereas in two tumor types, it served as both a protective and risk factor for progression‐free survival. These findings highlight the important role of KDM3B in predicting cancer outcomes and suggest that it could be a valuable biomarker.

Our findings indicate a substantial involvement of KDM3B in immune‐related IFN‐*α* and IFN‐*γ* pathways, as well as in other DEGs across various tumors. It appears that KDM3B may exert regulatory control over lymphocyte activity through epigenetic modifications within the tumor immune microenvironment [46]. This is further supported by its close association with ligand‐receptor interactions between malignant cells and immune cells [47]. Notably, in most tumors, there is a positive correlation between KDM3B expression and the infiltration of B cells, myeloid dendritic cells, macrophages, CD4^+^ T cells, and CD8^+^ T cells [48]. These findings suggest a potential influence of KDM3B on tumor development and prognosis through its impact on the tumor immune microenvironment. Additionally, our correlation analysis between KDM3B and pan‐cancer immunomodulators revealed a significant association of KDM3B expression with specific immunomodulatory genes.

In the context of immune regulation, immune checkpoints serve as crucial modulators on immune cells, particularly T cells, aiding in maintaining self‐tolerance and preventing autoimmunity [49, 50]. Cancer cells can exploit these checkpoints to evade immune detection and destruction [51]. Immune ICIs, which target checkpoint molecules such as CTLA4, PD‐1, and PD‐L1, have shown efficacy in releasing the immune system′s restraints and promoting antitumor responses [52]. Our study found notable associations between KDM3B expression and PD‐L1, PD‐1, and CTLA‐4 targets in various tumors. Although existing studies have not provided direct experimental evidence confirming that KDM3B directly regulates immune checkpoints (PD‐1, PD‐L1, and CTLA‐4), and the differences in its regulatory mechanisms across different tumor types remain unclear, our findings revealed an association between KDM3B and the well‐established predictive biomarkers for immunotherapy TMB and MSI. Studies have confirmed that TMB and MSI are closely correlated with the efficacy of ICBs [9, 53, 54]. Tumors with MSI‐H (dMMR) and TMB‐H tend to generate neoantigens and activate T‐cell immune responses [55]. However, tumor cells can suppress T‐cell activity and achieve immune escape by upregulating PD‐L1 and other immune checkpoint molecules, thus rendering patients with such tumors more sensitive to ICB therapy [56]. Prostate cancer studies have verified that both can serve as predictive indicators for ICB efficacy [57]. Could KDM3B affect the functions of immune checkpoints (PD‐1, PD‐L1, and CTLA‐4) through its regulation of TMB and MSI? Future experiments can be conducted to verify the binding ability and regulatory effects of KDM3B on TMB, MSI, and immune checkpoints, clarify their regulatory relationships, and thereby provide a novel theoretical basis for combined tumor immunotherapy.

Our study has several limitations that should be considered. In this investigation, KDM3B was studied within the context of cancer as a whole, based on the analysis of mRNA and protein expression levels in tumor sample datasets. Pan‐cancer analysis often relies on large‐scale gene expression data from both cancerous and normal tissues. Although publicly available databases such as TCGA and GTEx provide valuable cancer‐related information, these datasets may not encompass all cancer types, and their sample sizes may be insufficient for comprehensive analysis. Moreover, limitations associated with data types also pose a significant challenge. For instance, RNA‐seq data alone may not be sufficient for a comprehensive analysis of gene expression levels, as it is essential to consider other types of data, such as genomic variations and protein expression, to obtain a more complete understanding. To enhance the reliability of the database analysis, we conducted preliminary experiments to validate the credibility of the results. However, the mechanism by which KDM3B influences tumorigenesis and progression remains to be further elucidated based on the experiments conducted.

This comprehensive pan‐cancer analysis of KDM3B expression is the first of its kind, integrating KDM3B expression, subcellular localization, prognostic value, variations across different pathological stages, as well as KEGG and GO enrichment analyses, immune infiltration analysis, immune regulatory genes, TMB, and MSI in a diverse cohort of cancer samples. It positions KDM3B as a potentially vital therapeutic indicator and drug target, paving the way for advancements in cancer treatment strategies.

**Nomenclature**
GBM: glioblastoma multiforme
LGG: brain lower grade glioma
GBMLGG: glioblastoma‐lower grade glioma
ESCA: esophageal carcinoma
STES: stomach and esophageal carcinoma
COAD: colon adenocarcinoma
READ: rectum adenocarcinoma
STAD: stomach adenocarcinoma
KIRC: kidney renal clear cell carcinoma
LIHC: liver hepatocellular carcinoma
WT: wilms tumor
PAAD: pancreatic adenocarcinoma
TGCT: testicular germ cell tumors
ALL: acute lymphoblastic leukemia
LAML: acute myeloid leukemia
ACC: adrenocortical carcinoma
CHOL: cholangiocarcinoma
UCEC: uterine corpus endometrial carcinoma
CESC: cervical squamous cell carcinoma and endocervical adenocarcinoma
LUAD: lung adenocarcinoma
LUSC: lung squamous cell carcinoma
SKCM: skin cutaneous melanoma
BLCA: bladder urothelial carcinoma
THCA: thyroid carcinoma
OV: ovarian serous cystadenocarcinoma
UCS: uterine carcinosarcoma
KICH: kidney chromophobe
DLBC: diffuse Large B‐cell lymphoma
HNSC: head and neck squamous cell carcinoma
PRAD: prostate adenocarcinoma
BRCA: breast invasive carcinoma

## Author Contributions

L.Z. and X.P. interpreted and analyzed the data of the tumor public database. H.Y. and X.F. are responsible for the manuscript preparation. Z.Y. is responsible for resources, data curation, supervision, funding acquisition, and project administration.

## Funding

This study was supported by the National Natural Science Foundation of China (82000166), Joint Funds for the Innovation of Science and Technology, Fujian Province (2021Y9184), the Province‐Level Special Subsidy Funds for Health in Fujian Province (Fujian Finance Index(2023)830), the Fujian Provincial Natural Science Foundation of China (2022J011032, 2022J011063), and the Research Fund Project of Fujian Maternal and Child Health Hospital (YCXM20‐11).

## Conflicts of Interest

The authors declare no conflicts of interest.

## Data Availability

The data that support the findings of this study are available in the UCSC Xena at https://xenabrowser.net/datapages/. These data were derived from the following resources available in the public domain: The Gene Expression Profiling (http://gepia2.cancer-pku.cn/#dataset), cBioPortal (http://www.cbioportal.org), the Human Protein Atlas (https://www.proteinatlas.org/), the STRING database (https://string-db.org/), Hallmark gene set, (https://www.gsea-msigdb.org/gsea), and the TIMER2.0 database (http://timer.cistrome.org/). Basic experimental data further inquiries can be directed to the corresponding author.
